# Synergistic Effect of Graphene Oxide and Mesoporous Structure on Flame Retardancy of Nature Rubber/IFR Composites

**DOI:** 10.3390/ma11061005

**Published:** 2018-06-13

**Authors:** Na Wang, Miao Zhang, Ping Kang, Jing Zhang, Qinghong Fang, Wenda Li

**Affiliations:** 1 Sino-Spanish Advanced Materials Institute, Shenyang University of Chemical Technology, Shenyang 110142, China; iamzhangmiao@163.com; 2 Liaoning Provincial Key Laboratory of Rubber & Elastomer, Shenyang 110142, China; kangping@syuct.edu.cn (P.K.); zhangjingcszx@syuct.edu.cn (J.Z.); 17824917582@163.com (Q.F.); 3 IMDEA Materials Institute, C/Eric Kandel 2, Getafe, 28906 Madrid, Spain; wenda9011@163.com

**Keywords:** natural rubber, GO-NH-MCM-41, graphene oxide, mesoporous, intumescent flame retardants

## Abstract

Aiming to improve the flame retardancy performance of natural rubber (NR), we developed a novel flame retardant synergistic agent through grafting of MCM-41 to graphene oxide (GO), named as GO-NH-MCM-41, as an assistant to intumescent flame retardants (IFR). The flame retardancy of NR/IFR/GO-NH-MCM-41 composites was evaluated by limited oxygen index (LOI), UL-94, and cone calorimeter test. The LOI value of NR/IFR/GO-NH-MCM-41 reached 26.3%; the UL-94 ratings improved to a V-0 rating. Moreover, the addition of GO-NH-MCM-41 decreased the peak heat release rate (PHRR) and the total heat release (THR) of the natural rubber composites. Furthermore, the addition of GO-NH-MCM-41 increased the thickness of char residue. The images of SEM indicated the char residue was more compact and continuous. The degradation of GO-NH-MCM-41-based NR composites was completed with a mass loss of 35.57% at 600 °C. The tensile strength and the elongation at break of the NR/IFR/GO-NH-MCM-41 composites were 13.9 MPa and 496.7%, respectively. The results of the rubber process analyzer (RPA) reached the maximum value, probably due to a better network of fillers in the matrix.

## 1. Introduction

Natural rubber (NR) is considered one of the most important thermoset polymers, widely used as an economic industrial raw material in many fields due to its outstanding mechanical properties, chemical resistance, and good insulation. Unfortunately, the high combustibility of NR has hindered wide applications [1]. Several methods have been attempted to strengthen the flame retardancy of NR. The intumescent flame retardants (IFR) system is an effective way to strengthen the flame retardancy properties of NR; one of the widely used IFR systems consists of ammonium polyphosphate (APP), pentaerythritol (PER), and melamine (MEL) [2]. It has some advantages such as low smoke, less toxicity, low loading, and no dropping behavior, etc. However, compared with halogen-containing flame retardants, IFR has some disadvantages in terms of flame retardant efficiency and thermal stability. For example, IFR is easy to migrate to the composite surface. The polar groups in IFR molecules such as –OH and –NH_4_ groups have poor compatibility with the non-polar polymer matrix and thus result in a decrease in the flame retardancy and the thermal stability. 

It is an effective approach to strengthen the flame retardancy and mechanical properties of flame retardant natural rubber (FRNR) with the introduction of synergist flame retardant [3]. The application of graphene oxide as a synergist in an NR system has become more and more popular. Graphene oxide (GO) can generate enormous insulating layers when the composite is heated, due to the fact that graphene oxide is composed of carbon atoms having two-dimensional crystal with only one atom layer. Due to its special tri-dimensional structure, it can increase the amount of char residue and improve the thermal stability of FRNR. The limiting oxygen index value increases with the addition of graphene oxide, and higher ratings are achieved in a UL-94 test [4]. However, graphene oxide shows a tendency of agglomeration in the matrix, which leads to the recreation of pristine graphite, due to the interactions of π electrons and Van der Waals forces [5]. This results in inefficient dispersion of GO in a polymer matrix, which has a negative influence on the mechanical properties of FRNR [6,7]. 

Mesoporous molecular sieves have attracted the interest of researchers because of their high specific surface area and pore volume, uniform pore size distribution, and abundant surface groups [8]. MCM-41 has the characteristics of uniform channel, orderly arrangement of six parties, and adjus aperture. In our previous work, MCM-41 was used as a reinforced filler to enhance the mechanical and thermal properties of polymer materials [9]. Numerous publications have been devoted to the preparation, characterization, and properties of polymer/mesoporous MCM-41 composites [10,11,12,13]. The NR/MCM-41 nanocomposite showed enhanced tensile modulus which was higher than that of neat NR [14]. MCM-41 and APP were used as the core of double-layered co-microencapsulation. MCM-41 provided many binding sites, which strengthened the intumescent char [15,16]. When we add GO-NH-MCM-41 as synergistic flame retardant into epoxy resin (EP), 2 wt. % GO-NH-MCM-41 shows the exfoliated nano-dispersion in the EP matrix and a 40.0% reduction of peak heat release rate (PHRR) in cone calorimeter test was observed in EP/GO-NH-MCM-41 when compared to EP/GO. The structure of GO-NH-MCM-41 is characterized by Fourier-transform infrared spectroscopy, transmission electron microscope and scanning electron microscopy [17]. In this paper, the synergistic mechanism of GO-NH-MCM-41 in FRNR is investigated. The effects of GO-NH-MCM-41 on flame retardancy properties of NR are measured by limiting oxygen index, UL-94 test, thermogravimetric analysis, and cone calorimeter test and mechanical properties were characterized by tensile test and rubber process analyzer (RPA). The synergistic mechanism of GO and MCM-41 are also investigated.

## 2. Materials and Methods 

### 2.1. Materials

The nano-sized mesoporous MCM-41 particle was prepared in our laboratory with a lateral size of 80–100 nm. The surface area and specific pore volume of the MCM-41 particles were 732 m^2^/g and 0.9 cm^3^/g; the lateral size of pore was only 3.6 nm. The nano-sized graphite was supplied by Beijing Deke Graphite Co., Ltd., Beijing, China. Sulfuric acid (H_2_SO_4_, 98%), potassium permanganate (KMnO_4_), hydrazine hydrate (85% aq), ammonia (25–28% aq), hydrogen peroxide (H_2_O_2_, 30% aq), hydrochloric acid (HCl, 37% aq, diluted to 5 wt. % before use) were used as received. NR SMR-20 was got by Hainan state farms Group Co., Ltd., Haikou, China. The commercial product APP (phase II, average degree of polymerization n > 1000, soluble in H_2_O < 0.5 mass %) was bought from Shifang Changfeng Chemical Co., Ltd., Chengdu, China. PER and MEL were supplied by Sinopharm Chemical Reagent Co., Ltd., Shenyang, China. The mass ratio of APP, PER, and MEL in the IFR mixture was 3:1:1. Carbon soot and age inhibitor 4010 were bought from Sheng Ao Chemical Co., Ltd., Tianjin, China. Analytical grade ZnO was provided by Dalian ZnO factory, Dalian, China. Accelerant CZ and tetramethylthiuram disulfide were purchased from Tianjin No.1 Organic Chemical Plant, Tianjin, China. Sulfur was purchased from Tong Chuang Chemical Co., Ltd., Taizhou, China.

### 2.2. Methods

#### 2.2.1. Preparation of GO

GO was obtained from natural graphite by a modified Hummers method [18]. In brief, the graphite powder was treated with a solution containing a mixture of H_2_SO_4_, K_2_S_2_O_8,_ and P_2_O_5_ at 80 °C to yield preoxidized graphite. Then, it was added into cold H_2_SO_4_, followed by KMnO_4_, slowly. Distilled water and H_2_O_2_ terminated the reaction. The system was filtered and washed with HCl solution and dried to get brown solid. The solid was exfoliated for about 30 min in distilled water, in order to form stable GO dispersion (0.2 wt. %). 

#### 2.2.2. Preparation of MCM-41–NH2

(3-aminopropyl) triethoxysilane (APTES) (1.024 mL) was used to modify MCM-41, the APTES solution was refluxed in toluene at 105 °C for 120 min. This step was repeated three times to obtain MCM-41-NH_2_. 

#### 2.2.3. Preparation of GO-NH-MCM-41

**Figure 1:**
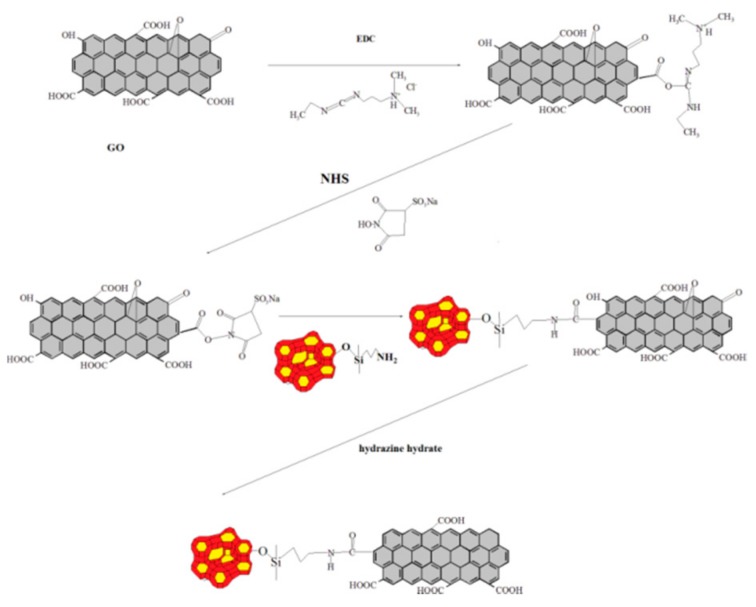
Reaction process of GO-NH-MCM-41.

A scheme for the synthesis of GO-NH-MCM-41 is shown in Figure 1. Firstly, 1-ethyl-3-(3-dimethyl aminopropyl) carbodiimide (EDC) (0.035 g) and N-hydroxysuccinimide (NHS) (0.023 g) were added into 20 mL distilled water. 11 mL of the above activated solution were slowly added into 500 mL 0.2 wt. % GO dispersion for about 2.5 h. Then, MCM-41-NH_2_ (5 g) was added into the above reaction mixture and stirred for 2 h at 40 °C. The resulting product GO-NH-MCM-41 was washed with distilled water and methanol for 2–3 times.

GO-NH-MCM-41 was suspended in water (400 mL) with ultrasonication and strong stirring. Reduction was carried out at 80 °C in the presence of hydrazine hydrate (the weight of GO was as same as hydrazine hydrate). Strong stirring and ultrasonication should be carried throughout the whole preparation process. GO-NH-MCM-41 powder was isolated via filtration and washed with distilled water four times, and dried to remove residual solvents at 60 °C for 24 h.

#### 2.2.4. Preparation of FRNR Composites

FRNR composites were synthesized by different proportions of IFR agents with NR by a two-roll mill. The compositions of FRNR composites are listed in Table 1. The fixed values of carbon soot, zinc oxide, stearic acid, sulfur, age inhibitor 4010, accelerant CZ, electric insulating oil, and tetramethylthiuram disulfide were 35, 5, 5, 4, 1.2, 1, 1, 0.8 and 0.35 phr [19]. All samples were vulcanized in a temperature of 145 °C at certain pressure. The optimum cure time t90 was determined by a GT-M2000-A rheometer (GaoTie Limited Co., Taiwan). All specimens were cut into vulcanized sheets at room temperature for 24 h before next test.

#### 2.2.5. Characterization

The Fourier transform infrared spectra of samples were measured with a Nicolet MAGNA-IR 560 with 4 cm^−1^ resolution. Limiting oxygen index (LOI) data were evaluated at room temperature by an oxygen index instrument (JF-3) which was provided by Jiangning Analysis Instrument Co., Jiangning, China. According to the GB/T 10707-2008 standard, all samples were tested five times. The vertical burning tests (UL-94 test) were measured with a CZF-3 type instrument, which was provided by Jiangning Analysis Instrument Co., Jiangning, China, according to the GB/T 10707-2008 standard. Room temperature tensile tests were measured on an Instron 1211 testing machine, GaoTie Limited Co., Taizhong, China. according to the GB/T528-1998 standard with a crosshead speed of 500 mm/min. The cone calorimeter tests were measured on a Fire Testing Technology cone calorimeter. The size of square specimens was 100 mm × 100 mm × 4 mm, each simple was tested twice at a heat flux of 35 kW/m^2^, according to ISO 5660 standard procedures without “frame and grid”. The exhaust flow was 24 L/s. The scanning electron microscopy (SEM) images of burnt samples were obtained by scanning electron microscope JEOL JSM-6360LV, Japan Electronics Co., Tokyo, Japan. Thermal stability of NR composites was performed on a Perkin-Elmer TGA 7 thermal analyzer, PerkinElmer Limited Co., Shanghai, China. The heating rate was set at 10 °C/min from room temperature to 700 °C under nitrogen with a flow rate of 80 mL/min. Curing was performed on rubber process analyzer RPA-8000 provided by GaoTie Limited Co., Taizhong, China, it was tested at 145 °C with a frequency of 100 cpm. Its strain amplitude was 0.5°.

## 3. Results and Discussion

### 3.1. Flame Retardancy

The flame retardancy properties and mechanical properties of composites were tested using standard tests. The data of the UL-94 ratings and LOI tests are showed in Table 2. The LOI value of NR was 18.2%, it had high flammability [20]. As an IFR was added into NR, the LOI value of the NR/IFR composite was increased to 22.4% with a V-1 rating. Meanwhile, when MCM-41 was used as the synergistic agent of IFR, the LOI values of NR/IFR/MCM-41 composites reached to 25.6% and achieved a V-0 rating. This can be explained by the fact that the MCM-41 reduced the amount of amorphous char in order to protect and strengthen the intumescent char [21]. When GO was used as synergistic agent, the LOI value of NR/IFR/GO composite reached to 24.3% and achieved a V-1 rating. This phenomenon could probably be explained by the fact that the presence of GO could facilitate the formation of strong char layers, which could delay weight loss caused by decomposition [22]. In addition, the LOI value of the FRNR composite containing GO-NH-MCM-41 was 26.3% and it showed an obvious increase of 17.4% when compared to NR/IFR systems. It also increased the oxidative degradation temperature and decreased the oxidization heat. It confirmed that GO-NH-MCM-41 was an excellent flame retardant synergist in improving the flame retardancy of NR.

### 3.2. Mechanical Properties

The mechanical properties of FRNR composites are listed in Table 2. The tensile strength and elongation at break of NR were 18.9 MPa and 591.3%, whereas they were 9.5 MPa and 479.6% for NR/IFR composites, respectively. It was found that IFR can remarkably decrease its mechanical properties. The great difference in polarity between the NR matrix and IFR fillers led to incompatibility and poor dispersion [23]. In contrast, when MCM-41 and GO were used as flame retardant synergistic agents separately, the tensile strength and elongation at break increased. Due to the large specific surface area and massive binding sites of MCM-41 and GO, the polar groups present in synergists, such as the amine and hydroxy group, can form hydrogen bonds with the amine groups of intumescent flame retardant [24]. It dispersed well and formed a better interaction with the rubber matrix than IFR [25]. The maximum values of tensile strength and elongation at break of GO-NH-MCM-41 reached to 13.9 MPa and 496.7%, higher than the values of MCM-41 and GO. This result confirmed that the GO-NH-MCM-41 can improve not only flame retardant properties but also mechanical performance.

### 3.3. Cone Calorimetry

**Figure 2:**
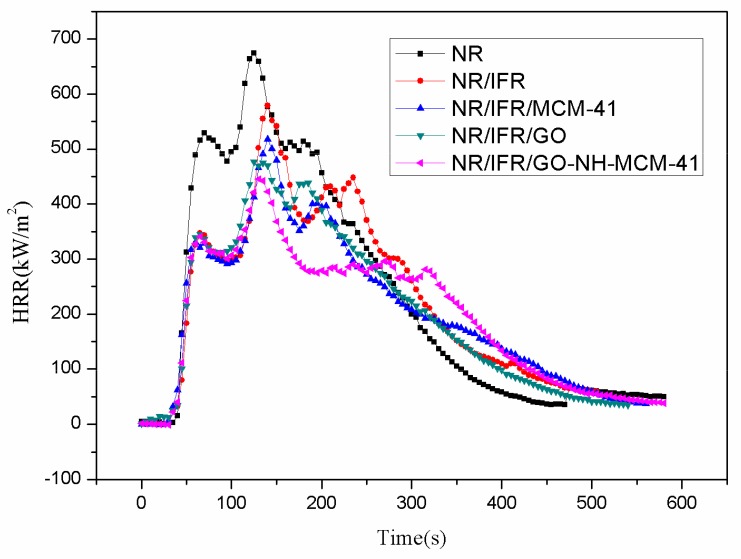
Heat release rate (HRR) curves of flame retardant natural rubber (FRNR) composites.

**Figure 3:**
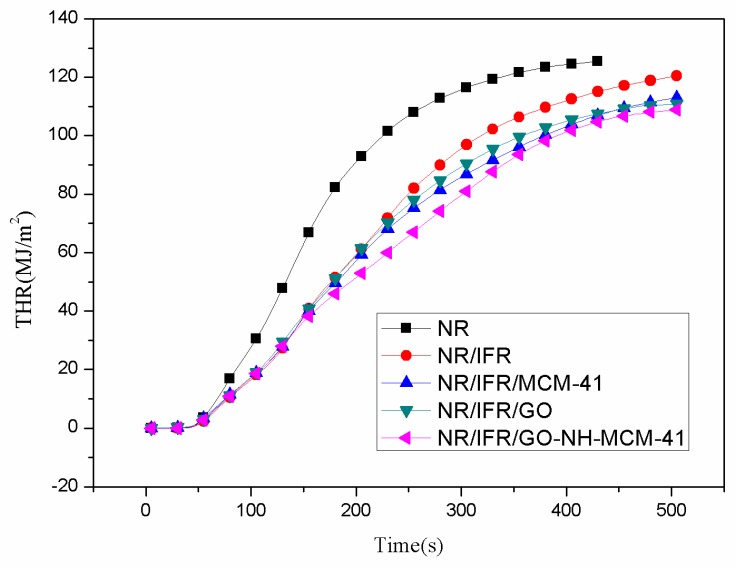
Total heat release (THR) curves of FRNR composites.

**Figure 4:**
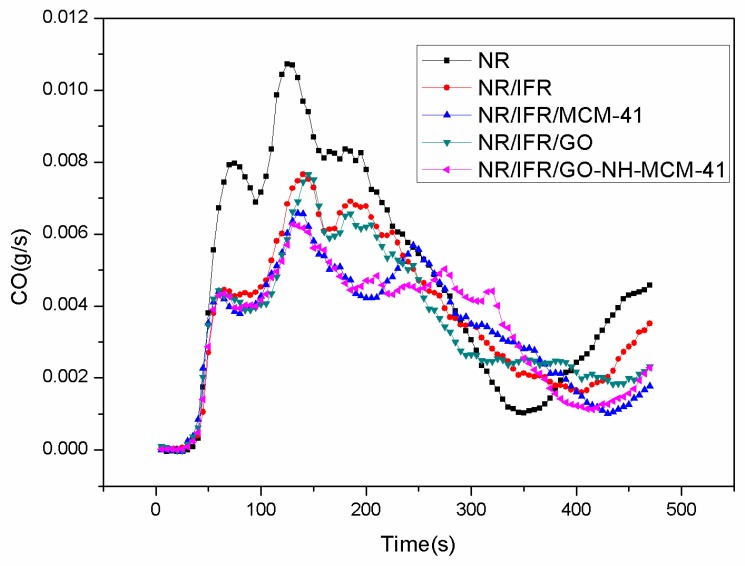
CO curves of FRNR composites.

**Figure 5:**
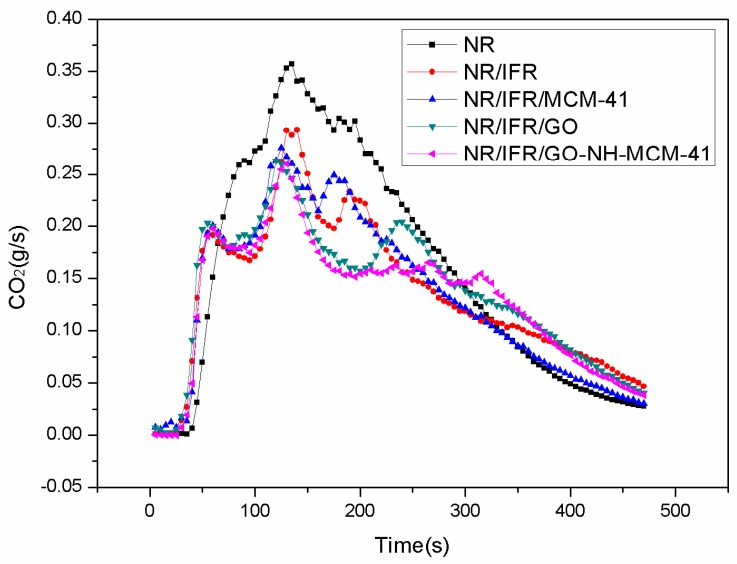
CO_2_ curves of FRNR composites.

Cone calorimetry is the most effective characterization of combustibility in real fire scenarios because it has a good correlation with actual fire disasters [26,27,28]. The images and data of the cone calorimeter test are shown in Figure 2, Figure 3, Figure 4 and Figure 5 and Table 3, respectively. In Figure 2, the heat release rate (HHR) curves of NR and IFR were double-peak pattern [29]. Because the rubber component was heated, its surface was cross-linked and carbonized and the carbon layers blocked oxygen and heat from outside [30]. At this time, combustion behavior was suppressed and the first peak appeared but as the combustion depth increased, the surface carbon layer was destroyed, the rubber under the carbon layers continued to crack, releasing more combustible gas, and total heat release (THR) and HRR reached the maximum peak value. Therefore, we can conclude that the burning of natural rubber was intense [31]. When IFR was added into NR, this situation changed, with the values of PHRR and THR decreasing by 23.3% and 10.5%. This phenomenon was due to the flame retardancy of IFR. The char layers were formed by IFR, which can block the exchange of oxygen and heat from inside and outside. The degree of combustion was controlled, but the char layers were not firm enough [32]. When MCM-41 and GO were used as synergistic agents, PHRR values decreased to 477 kW/m^2^ and 455 kW/m^2^, respectively. PHRR of NR/IFR/GO-NH-MCM-41 showed a decrease of 34% when compared to NR. In particular, THR, CO and CO_2_ yields of NR/IFR/GO-NH-MCM-41 composites decreased by 14%, 50% and 39% when compared with NR. From the above figures and tables we can notice that the THR and HHR of NR/IFR/GO and NR/IFR/MCM-41 decreased at a certain degree. Compared to NR/IFR, these decreases were not obvious. The flame retardant mechanisms of GO and MCM-41 were different [33]. Referring to the previously published literature [34], GO has an outstanding property of increasing the content of residual char. When it was used alone with IFR, the intumescent char layer was massive, but it was fragile, the barrier property was poor [35]. As to the MCM-41, the residual char added with MCM-41 had a better barrier property, but its volume was not enough, so the flame retardant NR/IFR/MCM-41 was worse than NR/IFR/GO-NH-MCM-41 [6]. When GO-NH-MCM-41 was used as flame retardancy, the synergistic effect was clear from the cone test. We can know from the HHR curves that after the maximum peak value the curve of NR/IFR/GO-NH-MCM-41 was more flat and lower than others and the curves of CO and CO_2_ also followed the phenomenon, illustrating that the combustion was smooth [36]. The residual char of NR/IFR/GO-NH-MCM-41 was compact and frim and its thickness was better than others. GO-NH-MCM-41 combined the advantages of GO and MCM-41.

### 3.4. Thermal Stability

**Figure 6:**
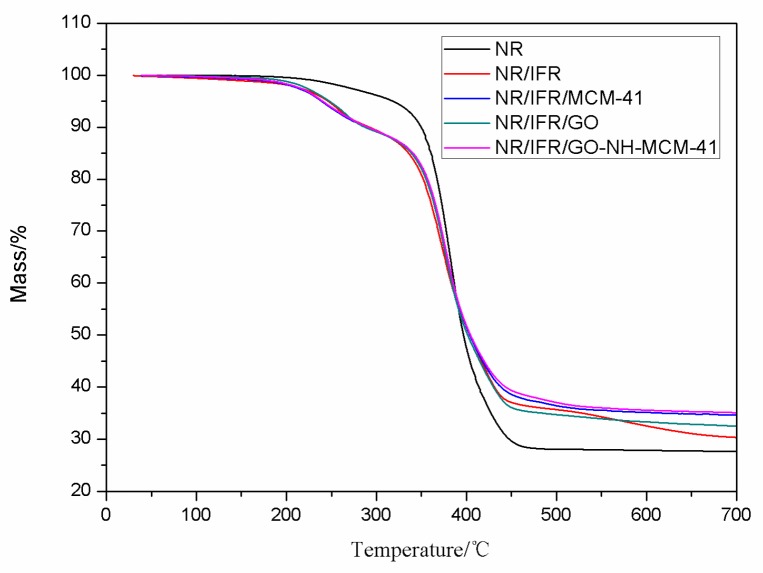
TG curves of natural rubber (NR) and FRNR composites.

**Figure 7:**
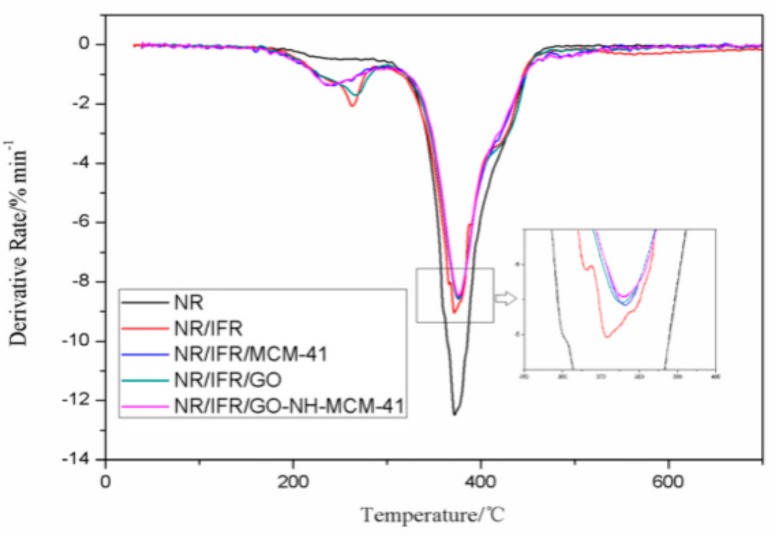
DTG curves of NR and FRNR composites.

TG was used to analyze the thermal degradation performance of NR composites. The related informations of TG are listed in Figure 6 and Figure 7, Table 4. The 10 wt. % weight loss temperature of NR/IFR was decreased to 292 °C, an approximately 16% reduction compared to that of pure NR, because the IFR had poor thermal stability, and could decompose at 200 °C [37]. PER produced ester polyols and the acid as a dehydrating agent. Ester polyols carbonized those produced molecules of water to decrease the temperature of environment and the acid had an esterification reaction with PER to form carbon layers. MEL and APP also produced noncombustible gas NH_3_, which expanded the carbon layers [38]. At the beginning of combustion, the char layers were too thin to prevent oxygen and heat from diffusing into the matrix. As the surface area of the combustion boundary increased, the combustion process developed more quickly. At the initial degradation, the intumescent char layers formed and the intumescent char layers lowered the diffusion rate of volatile combustible fragments, so its mass loss rate decreased [39]. The degradation of NR/IFR/GO-NH-MCM-41 ended with a mass loss of 35.57%, which was higher than other samples. It was found that FRNR composites achieved better thermal stability in the presence of the intumescent flame retardants, because it could decompose into polyphosphoric acid, water gas, and ammonia gas at high temperatures. They might get coated at the surface of the matrix to prevent the heat from diffusing into the matrix, so that temperature gradient between char layers and matrix was increased [40]. This may suggest that the strength of residual char was attributed to the excellence of char forming and mechanical enhancement performance of GO-NH-MCM-41. GO had an excellent thermal property and expanded several hundred times along C–C axial when it was heated [41]. The residue of NR/IFR/GO-NH-MCM-41 was more continuous and compact than others, so the protective barrier char had an excellent property of preventing the degradation of the NR matrix.

### 3.5. Morphologies of Burnt Composites

**Figure 8:**
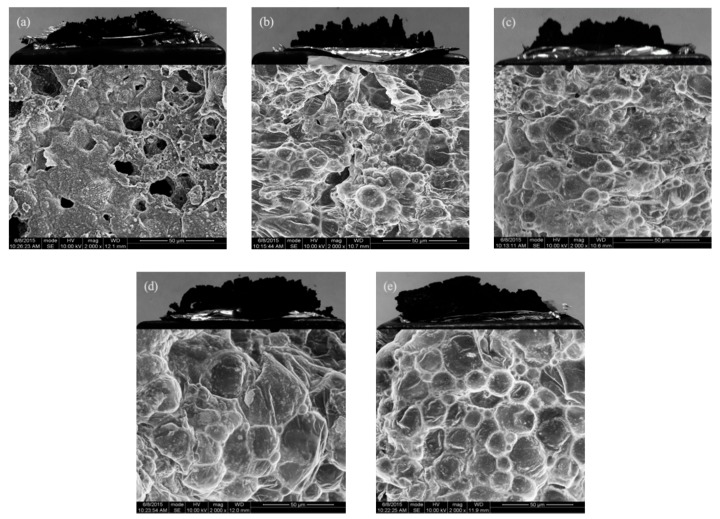
Morphology of burnt composites (**a**) NR; (**b**) NR/IFR; (**c**) NR/IFR/MCM-41; (**d**) NR/IFR/GO; (**e**) NR/IFR/GO-NH-MCM-41.

It was known that continuous and compact char layers can be seen as an excellent insulating barrier to heat and oxygen outside. We took SEM photos of the residual char, which were procured by an LOI test. As shown in Figure 8a, there was a loose structure that contained cracks and cavities appeared on the surface of NR residual char. In NR/IFR systems, some hollows could be seen in fractured char (Figure 8b) [42]. When MCM-41 and GO were used as synergistic agents, the char layer of NR systems (Figure 8c,d) became more compact with fewer holes when compared to that of NR/IFR systems. However, we could hardly find defects on the surface of char layers in NR/IFR/GO-NH-MCM-41 (Figure 8e). A compact and continuous char layer formed during the burning of the NR composites. In addition, we could also observe more uniform blisters in the image. It reflected that GO-NH-MCM-41 was effective in forming more compact char layers, because GO-NH-MCM-41 had good compatibility with the matrix and formed a network structure at high temperature in a fire hazard situation.

**Figure 9:**
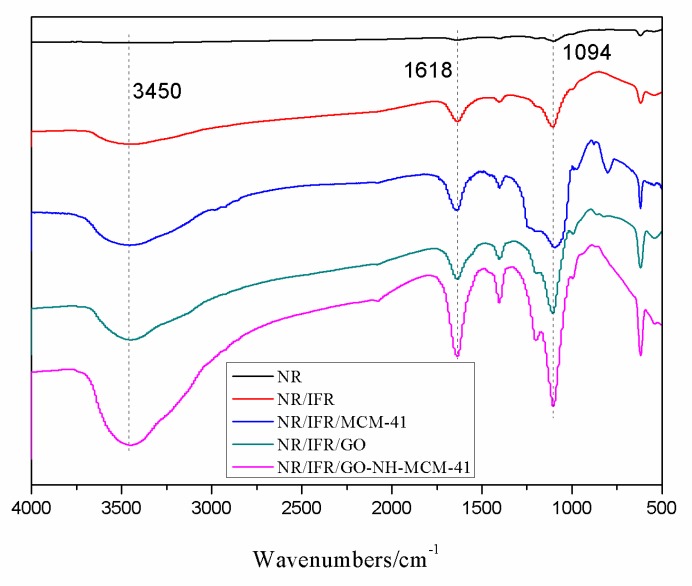
FTIR spectra of char residues of FRNR composites after burning.

In order to investigate the structure and compositions of char, FTIR spectra of char residues were recorded in Figure 9. The samples were procured by heating in a muffle furnace for 10 min at 600 °C. The absorption peak at 3450 cm^−1^ was attributed to the water molecule. The absorption peak at 1618 cm^−1^ revealed the presence of a multi-aromatic structure in the char residue [43]. NR composites showed a similar char structure due to the similar spectrum of pure NR. An intensive absorption peak at 1094 cm^−1^ was assigned to vibrations of the P–O–C group. It was found that the stretching vibration intensity of different chemical groups increased from top to bottom, which proved the increase in flame retardancy products [44]. The char layers, composed of multi-aromatic carbon and phosphorus-containing structures had a high thermal stability, so they can act as an effective barrier to prevent the matrix from decomposition at high temperature [45].

### 3.6. Rubber Process Analyzer (RPA) of FRNR Composites

**Figure 10:**
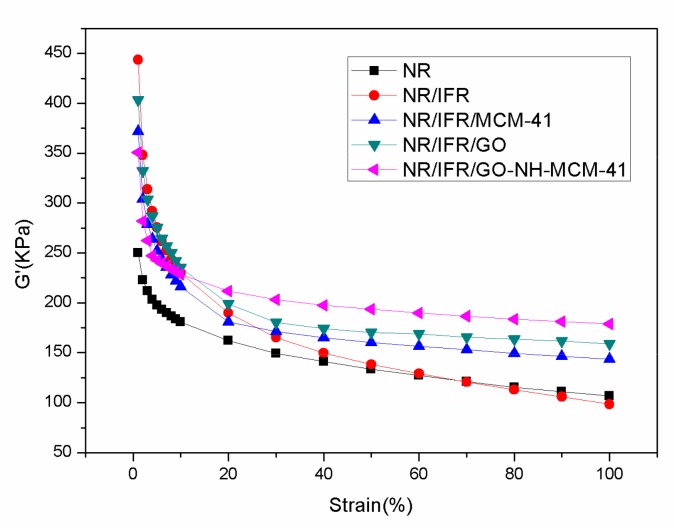
Dependence of *G′* on strain of NR and FRNR composites.

The dependence of *G′* on strain of FRNR composites are shown in Figure 10 and Table 5. The filler network was broken down at a small strain and this was generally called Payne effect [46]. Δ*G′* was often deemed as an important indicator for the strength of the filler network. Because the *G′* value decreased as the filler network strength reduced, it can be clearly seen from Figure 10 that all NR composites had a typical Payne effect [47]. In addition, the *G′* values of all composites decreased when the strain was 1%. At the same time, the values of *G′* decreased gradually as the strain increased. The *G′* almost reached the fixed value when the strain of unfilled rubber arrived at about 100%. From Table 5, it can be seen that the Δ*G′* values of NR composites decreased in the following order: NR/IFR > NR/IFR/GO > NR/IFR/MCM-41 > NR/IFR/GO-NH-MCM-41. Therefore, the strength of the filler network increased in the order of NR/IFR/GO-NH-MCM-41 > NR/IFR/MCM-41 > NR/IFR/GO > NR/IFR. This was because GO-NH-MCM-41 had an optimized compatibility with the matrix. As a result, NR/IFR/GO-NH-MCM-41 formed a better filler network in FRNR composites and thus GO-NH-MCM-41 had a positive effect in improving the mechanical properties of NR composites.

## 4. Conclusions

In conclusion, GO-NH-MCM-41 had outstanding flame retardancy properties. It increased the LOI values of FRNR composites from 22.4% to 26.3% and achieved a V-0 rating in UL-94 test. The PHRR, THR, CO yield, and CO_2_ yield of NR/IFR/GO-NH-MCM-41 composites decreased by 34%, 14%, 50% and 39% respectively. We can see from SEM images that the char layer of NR/IFR/GO-NH-MCM-41 composites was more compact and continuous. As a synergist, GO-NH-MCM-41 improved thermal stability of NR through increasing W600 of the residual char from 32% to 35%, which could delay weight loss and reduce the risk of fire hazard. It was mainly attributed to the synergistic effect of GO-NH-MCM-41and IFR.

The good mechanical properties of NR/IFR/GO-NH-MCM-41 were attributed to the addition of GO-NH-MCM-41. It had good compatibility with the matrix after modification and it can form a three-dimensional network structure with the matrix. The fine grafting between GO and MCM-41 resulted in a better dispersion in composites. Compared to NR/IFR, the values of tensile strength and elongation at break of NR/IFR/GO-NH-MCM-41 were increased from 9.5 MPa to 13.9 MPa and elongation at break of NR/IFR/GO-NH-MCM-41 were increased from 479.6% to 496.7%, which were all higher than NR/IFR/MCM-41 and NR/IFR/GO.

## Figures and Tables

**Table 1:** Ingredients of NR composites.

Samples	NR/phr	IFR/phr	MCM-41/phr	GO/phr	GO-MCM-41/phr
NR	100				
NR/IFR	100	40			
NR/IFR/MCM-41	100	39	1		
NR/IFR/GO	100	39		1	
NR/IFR/GO-MCM-41	100	39			1

**Table 2:** Flame retardancy and mechanical properties of flame retardancy natural rubber (FRNR) composites.

Samples	LOI/%	UL-94	Tensile Strength/MPa	Elongation at Break/%
NR	18.2	No rating	18.9	581.3
NR/IFR	22.4	V-1	9.5	479.6
NR/IFR/MCM-41	25.6	V-0	11.6	485.8
NR/IFR/GO	24.3	V-1	11.5	493.7
NR/IFR/GO-NH-MCM-41	26.3	V-0	13.9	496.7

**Table 3:** Cone data of FRNR composites.

Samples	PHRR (kW/m^2^)	THR (MJ/m^2^)	P-CO (g/s)	P-CO_2_ (g/s)
NR	675	128	0.0107	0.3567
NR/IFR	518	115	0.0077	0.2936
NR/IFR/MCM-41	477	113	0.0076	0.2759
NR/IFR/GO	455	124	0.0066	0.2636
NR/IFR/GO-NH-MCM-41	446	109	0.0063	0.2601

**Table 4:** TG data of FRNR composites.

Flame Retardant Composites	T_10%_/°C	T_20%_/°C	T_50%_/°C	W_600_/%
NR	349	367	396	27
NR/IFR	292	352	403	32
NR/IFR/MCM-41	289	356	403	35
NR/IFR/GO	287	355	401	33
NR/IFR/GO-MCM-41	290	356	404	35

**Table 5:** Δ*G′* values.

Samples	Δ*G′*/MPa
NR	1.18
NR/IFR	3.45
NR/IFR/MCM-41	2.28
NR/IFR/GO	2.45
NR/IFR/GO-NH-MCM-41	1.72

## References

[1] Carli L.N., Roncato C.R., Zanchet A., Mauler R.S., Giovanela M., Brandalise R.N., Crespo J.S. (2011). Characterization of natural rubber nanocomposites filled with organoclay as a substitute for silica obtained by the conventional two-roll mill method. Appl. Clay Sci..

[2] Rabe S., Chuenban Y., Schartel B. (2017). Exploring the modes of action of phosphorus-based flame retardants in polymeric systems. Materials.

[3] Iqbal S.S., Inam F., Iqbal N., Jamil T., Bashir A., Shahid M. (2016). Thermogravimetric, differential scanning calorimetric, and experimental thermal transport study of functionalized nanokaolinitedoped elastomeric nanocomposites. J. Therm. Anal. Calorim..

[4] Rafi M., Samiey B., Cheng C.H. (2018). Study of adsorption mechanism of congo red on graphene oxide/PAMAM nanocomposite. Materials.

[5] Wang X., Song L., Yang H.Y., Xing W.Y., Kandola B., Hu Y. (2012). Simultaneous reduction and surface functionalization of graphene oxide with POSS for reducing fire hazards in epoxy composites. J. Mater. Chem..

[6] Chen H.D., Wang J.H., Ni A.Q., Ding A.X., Han X., Sun Z.H. (2018). The Effects of a Macromolecular Charring Agent with Gas Phase and Condense Phase Synergistic Flame Retardant Capability on the Properties of PP/IFR Composites. Materials.

[7] Wu K., Zhang Y., Hu W., Lian J., Hu Y. (2013). Influence of ammonium polyphosphate microencapsulation on flame retardancy, thermal degradation and crystal structure of polypropylene composite. Compos. Sci. Technol..

[8] Wang Z., Liu Y., Li J. (2016). Preparation of nucleotide-based microsphere and its application in intumescent flame retardant polypropylene. J. Anal. Appl. Pyrolysis.

[9] Wang N., Shao Y., Shi Z., Zhang J., Li H. (2008). Preparation and characterization of epoxy composites filled with functionalized nano-sized MCM-41 particles. J. Mater. Sci..

[10] Wang N., Fang Q.H., Chen E.F., Shaohang J., Shao Y. (2010). Preparation and characterization of polypropylene composites filled with different structured mesoporous particles. J. Compos. Mater..

[11] Wang N., Gao N., Fang Q.H., Chen E.F. (2011). Compatibilizing effect of mesoporous fillers on the mechanical properties and morphology of polypropylene and polystyrene blend. Mater. Des..

[12] Wang N., Wu Y.X., Zhang J., Ma C., Chen E.F. (2012). Compatibilizing effect of MCM-41 and PP-g-MAH on the mechanical and thermal analyzer of PP/PS blends. Adv. Mater. Res..

[13] Wang N., Fang Q.H., Zhang J., Chen E.F., Zhang X.B. (2011). Incorporation of nano-sized mesoporous MCM-41 material used as fillers in natural rubber composite. Mater. Sci. Eng. A.

[14] Wang N., Zhang J., Fang Q.H., Hui D. (2013). Influence of mesoporous fillers with PP-g-MA on flammability and tensile behavior of polypropylene composites. Compos. Part B.

[15] Wang N., Gao N., Jiang S., Fang Q.H., Chen E.F. (2011). Effect of different structure MCM-41 fillers with PP-g-MA on mechanical properties of PP composites. Compos. Part B.

[16] Wang N., Mi L., Wu Y., Zhang J., Fang Q. (2014). Double-layered co-microencapsulated ammonium polyphosphate and mesoporous MCM-41 in intumescent flame-retardant natural rubber composites. J. Therm. Anal. Calorim..

[17] Li Z., González A.J., Heeralal V.B., Wang D.Y. (2018). Covalent assembly of MCM-41 nanospheres on graphene oxide for improving fire retardancy and mechanical property of epoxy resin. Compos. Part B.

[18] Hummers W.S., Offeman R.E. (1958). Preparation of graphitic oxide. J. Am. Chem. Soc..

[19] Rostamizadeh S., Azad M., Shadjou N., Hasanzadeh M. (2012). (α-Fe_2_O_3_)-MCM-41-SO_3_H as a novel magnetic nanocatalyst for the synthesis of N-aryl-2-amino-1,6-naphthyridine derivatives. Catal. Commun..

[20] Liu H., Wang X., Wu D. (2015). Preparation, isothermal, kinetics, and performance of a novel epoxy thermosetting system based on phosphazene-cyclomatrix network for halogen-free flame retardancy and high thermal stability. Thermochim. Acta.

[21] Huang T., Lu R.G., Su C., Wang H., Guo Z., Liu P., Huang Z., Chen H., Li T. (2012). Chemically modified graphene/polyimide composite films based on utilization of covalent bonding and oriented distribution. ACS Appl. Mater. Interfaces.

[22] Janowska G., Kucharska-Jastrząbek A., Rybiński P., Wesołek D., Wójcik I. (2010). Flammability of diene rubbers. J. Therm. Anal. Calorim..

[23] Wendels S., Chavez T., Bonnet M., Salmeia K.A., Gaan S. (2017). Recent Developments in Organophosphorus Flame Retardants Containing P-C Bond and Their Applications. Materials.

[24] Maciejewska M. (2016). Thermal properties of TRIM–GMA copolymers with pendant amine groups. J. Therm. Anal. Calorim..

[25] Chen X., Jiao C., Li S., Hu Y. (2013). Preparation and properties of a single molecule intumescent flame retardant. Fire Saf. J..

[26] Yang R., Ma B., Zhao H., Li J. (2016). Preparation, thermal degradation, and fire behaviors of intumescent flame retardant polypropylene with a charring agent containing pentaerythritol and triazine. Ind. Eng. Chem. Res..

[27] Liu Z., Dai M., Zhang Y., Gao X., Zhang Q. (2016). Preparation and performances of novel waterborne intumescent fire retardant coatings. Prog. Org. Coat..

[28] Xie H., Lai X., Zhou R., Li H., Zhang Y., Zeng X., Guo J. (2015). Effect and mechanism of n-alkoxy hindered amine on the flame retardancy, uv aging resistance and thermal degradation of intumescent flame retardant polypropylene. Polym. Degrad. Stab..

[29] Cao K., Wu S.L., Qiu S.L., Li Y., Yao Z. (2012). Synthesis of N-alkoxy hindered amine containing silane as a multifunctional flame retardant synergist and its application in intumescent flame retardant polypropylene. Ind. Eng. Chem. Res..

[30] Lai X., Yin C., Li H., Zeng X. (2015). Synergistic effect between silicone-containing macromolecular charring agent and ammonium polyphosphate in flame retardant polypropylene. J. Appl. Polym. Sci..

[31] Su X., Yi Y., Tao J., Qi H., Li D. (2014). Synergistic effect between a novel triazine charring agent and ammonium polyphosphate on flame retardancy and thermal behavior of polypropylene. Polym. Degrad. Stab..

[32] Reddy K.R., Kumar B., Rana S., Tevtia A.K., Singh R.P. (2007). Synthesis and characterization of hindered amine light stabilizers based on end functionalization of polypropylene. J. Appl. Polym. Sci..

[33] Xie H., Lai X., Li H., Zeng X. (2016). Synthesis of a novel macromolecular charring agent with free-radical quenching capability and its synergism in flame retardant polypropylene. Polym. Degrad. Stab..

[34] Sun H.Q., Liu S.Z., Zhou G., Ang H.M., Tadé M.O., Wang S.B. (2012). Reduced graphene oxide for catalytic oxidation of aqueous organic pollutants. ACS Appl. Mater. Interfaces.

[35] Toldy A., Niedermann P., Pomázi Á., Marosi G., Szolnoki B.C. (2017). Flame retardancy of carbon fibre reinforced sorbitol based bioepoxy composites with phosphorus-containing additives. Materials.

[36] Chen C.C., Li Z., Shi L., Cronin S.B. (2015). Thermoelectric Transport across Graphene/Hexagonal Boron Nitride/Graphene Heterostructures. Nano Res..

[37] Xu Z.Z., Huang J.Q., Chen M.J., Tan Y., Wang Y.Z. (2013). Flame retardant mechanism of an efficient flame-retardant polymeric synergist with ammonium polyphosphate for polypropylene. Polym. Degrad. Stab..

[38] Chen W., Fu X., Ge W., Xu J., Jiang M. (2014). Microencapsulation of bisneopentyl glycol dithiopyrophosphate and its flame retardant effect on polyvinyl alcohol. Polym. Degrad. Stab..

[39] Dang L., Nai X., Dong Y., Li W. (2017). Functional group effect on flame retardancy, thermal, and mechanical properties of organophosphorus-based magnesium oxysulfate whiskers as a flame retardant in polypropylene. RSC Adv..

[40] Zhang T., Yan H., Wang L., Fang Z. (2013). Controlled formation of self-extinguishing intumescent coating on ramie fabric via layer-by-layer assembly. Ind. Eng. Chem. Res..

[41] Altarawneh M., Dlugogorski B.Z. (2014). Mechanism of thermal decomposition of tetrabromobisphenol A (TBBA). J. Phys. Chem. A.

[42] Altarawneh M., Dlugogorski B.Z. (2014). Thermal decomposition of 1,2-Bis(2,4,6-tribromophenoxy)ethane (BTBPE), a novel brominated flame retardant. Environ. Sci. Technol..

[43] Cayla A., Rault F., Giraud S., Salaün F., Fierro V., Celzard A. (2016). PLA with intumescent system containing lignin and ammonium polyphosphate for flame retardant textile. Polymers.

[44] Yang S., Wang J., Huo S., Wang M., Wang J. (2015). Preparation and flame retardancy of a compounded epoxy resin system composed of phosphorus/nitrogen-containing active compounds. Polym. Degrad. Stab..

[45] Xiao L., Sun D.C., Niu T.L., Yao Y.W. (2014). Syntheses of two dopo-based reactive additives as flame retardants and co-curing agents for epoxy resins. Phosphorus Sulfur Silicon Relat. Elem..

[46] Xie J., Zhu Y., Bian F., Liu C. (2017). Dynamic recovery and recrystallization mechanisms during ultrasonic spot welding of Al-Cu-Mg alloy. Mater. Charact..

[47] Robertson C.G., Lin C.J., Bogoslovov R.B., Rackaitis M., Sadhukhan P., Quinn J.D., Roland C.M. (2011). Reinforcement, and glass transition effects in silica-filled styrene-butadiene rubber. Rubber Chem Technol..

